# How Healthcare Utilization Due to Dizziness and Vertigo Differs Between Older and Younger Adults

**DOI:** 10.3389/fmed.2022.852187

**Published:** 2022-02-16

**Authors:** Tino Prell, Sigrid Finn, Hubertus Axer

**Affiliations:** ^1^Department of Geriatrics, Halle University Hospital, Halle, Germany; ^2^Department of Neurology, Jena University Hospital, Jena, Germany; ^3^Center for Vertigo and Dizziness, Jena University Hospital, Jena, Germany

**Keywords:** dizziness, vertigo, healthcare utilization, older age, hospitalization

## Abstract

**Background:**

Vertigo and dizziness are common in older adults. We describe self-reported healthcare utilization because of dizziness and vertigo in older adults attending a tertiary care specialized vertigo center.

**Methods:**

Data from 765 patients (45% were ≥60 years old) with chronic dizziness and vertigo who attended a daycare multimodal treatment program were recorded. Data included sociodemographic parameters, dizziness-related characteristics, the Body Sensations Questionnaire (BSQ), the Agoraphobic Cognitions Questionnaire (ACQ), and the Hospital Anxiety and Depression Scale (HADS). Also, healthcare utilization, including (1) physician and clinical services, (2) hospitalizations in the year before consulting the vertigo center, (3) prescription of drugs and other professional services were included. Descriptive statistics, exploratory data analysis, and regression models were used.

**Results:**

Intensity of dizziness was similar in both age groups, however, distress due to dizziness was more severe in younger persons. Dizziness symptoms lasted longer in older adults than in younger persons. Older adults had a somatic diagnosis (74.6 vs. 35.0%) more frequently and reported more falls (37.2 vs. 28.5%) than younger individuals. Anxiety about bodily sensations was higher in younger patients (mean BSQ1 = 9.33 ± 5.6) than in older patients (mean BSQ1 = 6.72 ± 5.4). Older persons had fewer depressive symptoms (mean HADS depression = 5.8 ± 3.6 vs. 6.5 ± 4.1) and less anxiety (mean HADS anxiety = 5.7 ± 3.7 vs. 7.8 ± 4.1) than younger individuals. Younger people were more frequently hospitalized (24.4%) than older adults (16.3%) in the year before consulting the vertigo center. Also, younger patients and patients with non-somatic etiologies had considerably more consultations with healthcare providers than older patients. Older adults received less medication (50.3%), less physiotherapy (41%), and less psychological therapy (11.6%) for vertigo than younger people (59.7, 52.2, 20.4%, respectively).

**Conclusion:**

Age-associated differences in healthcare utilization were defined in selected patients attending a specialized vertigo center. Since dizziness is frequently a heterogeneous disorder requiring interdisciplinary care, its diagnostic and therapeutic work-up must be improved for older patients with dizziness.

## Introduction

Vertigo and dizziness have a high lifetime prevalence of about every fifth adult suffering from dizziness or vertigo (1). Data on the lifetime prevalence of dizziness and vertigo show an increasing prevalence of 30% to 50% in older people (2–5). In older patients, multifactorial, degenerative, and chronic causes of vertigo/dizziness prevail (6, 7). Older adults with dizziness often suffer from multisensory deficits, such as central vertigo bilateral vestibulopathy, and benign paroxysmal positional vertigo (8, 9). Especially in older individuals, vertigo and dizziness contribute to physical inactivity (10), social isolation, depression (11), dizziness-associated disabilities (12), and a higher risk of falls (13, 14). Therefore, dizziness and vertigo *per-se* and commonly associated psychiatric comorbidities, such as anxiety or depression, can cause increased healthcare utilization (15), incurring a high economic burden (16, 17). It is, therefore, necessary to identify factors that influence a person's use of healthcare services.

In general, healthcare services can be used to diagnose or treat disorders, improve or maintain function, or obtain information about health status. Healthcare utilization depends on the need for care, the will to obtain care, and access to care (18–20). Among other, healthcare utilization includes the prescription of drugs and hospital, physician, clinical, and nursing care services (21).

We describe self-reported healthcare utilization because of dizziness in older adults before attending a specialized vertigo center. We aimed to (1) describe differences for consulting healthcare providers, hospitalizations, and therapy in younger and older adults, (2) explore if these differences are associated with age or can be attributed to other factors.

## Methods

### Participants

Overall, 765 patients with chronic dizziness and vertigo attended a daycare multimodal treatment program (22) between June 2013 and September 2017 in the Center for Vertigo and Dizziness of Jena University Hospital. The Center for Vertigo and Dizziness is a tertiary care outpatient project consisting of neurology, ear, nose, and throat (ENT), and physiotherapy departments. The center provides interdisciplinary diagnoses of patients with chronic vertigo and dizziness and day care multimodal therapy (23). Requirements for participation in this outpatient treatment program were: Chronic dizziness and vertigo (i.e., symptoms have persisted for at least 3months or attacks have recurred at least 5 days per month), physical independence (i.e., walking independently) and no cognitive limitations that may affect the activities of daily living.

The study was approved by the local ethics committee (Ethics Committee of the Friedrich-Schiller-University Jena, Number 5426-02/18), and written informed consent for study participation was obtained from all patients.

### Assessments

We assessed the following self-reported sociodemographic and vertigo- and dizziness-related data on the first day of the treatment: age (metric; years), gender (binominal; male/female), a rough description of symptoms (binominal; the presence or absence of attacks, permanent dizziness/vertigo, or falls in the last 12 months), and the duration of symptoms (multinomial, ≤ 2 months, 2–6 months, 6–12 months, 1–2 years, 3–5 years, >5 years). In the last 3 months, the intensity of vertigo/dizziness and the distress due to vertigo/dizziness were quantified using a visual analog scale from 0 (no distress) to 10 (maximal distress). As defined at our center, advanced neurologists and vertigo specialists medically diagnosed and psychologically evaluated every patient.

The Body Sensations Questionnaire (BSQ) and the Agoraphobic Cognitions Questionnaire (ACQ) were also administered via self-report. The BSQ consists of 17 items measuring anxiety about bodily sensations on a 5-point Likert scale and comprises two questionnaires: the BSQ1 assesses the amount of fear and the BSQ2 measures how often the sensations are experienced when the patient feels anxious or tense (24). The ACQ is a 14-item questionnaire that measures maladaptive thoughts about the potential for catastrophic consequences of anxiety or panic. Cronbach's alphas of both questionnaires were high in our sample (BSQ1: α = 0.85; BSQ2 α = 0.94; ACQ: α = 0.81). Earlier studies used the ACQ and BSQ to evaluate patients with dizziness/vertigo (25, 26). The Hospital Anxiety and Depression Scale (HADS) was used to estimate anxiety and depression (27) and evaluate general psychological distress in patients with dizziness (28).

The different types of healthcare utilization were assessed according to the Centers for Medicare & Medicaid Services (CMS) categories via self-report (29): (1) physician and clinical services, (2) hospitalizations in the year before consulting the vertigo center, (3) prescription of drugs, and other professional services (physiotherapy). The utilization of “physician and clinical services” included consultations with a physiotherapist/chiropractor, alternative practitioner, general practitioner, ENT specialist, orthopedist, neurologist, psychiatrist, psychologist, or emergency department physician.

### Statistics

All data were analyzed with the Statistical Package for the Social Sciences software (version 25.0; IBM Corporation, USA) and R (R Foundation for Statistical Computing, Vienna, Austria) version 3.6.2 or JASP 0.16. Values are the mean and standard deviation (SD) or numbers and percentages. Normal distribution was determined using the Shapiro–Wilk test. Missing data were treated according to the pairwise deletion process.

First, the cohort was analyzed using descriptive statistics. Differences between younger (<60 years old) and older persons (≥60 years old) were determined by the *t*-test, *U*-test, and Chi-square test.

Elastic net regularization and binominal logistic regressions (backward selection) were used to determine the association between healthcare utilization and clinical variables (after excluding multicollinearity and autocorrelation) (30). Elastic net regularization generated parsimonious models, which are easier to interpret. Variable selection was performed by shrinking the parameters toward zero and attenuating overfitting, a well-known problem when applying regression models with a large number of predictors. Tenfold cross-validation was performed to choose the best model with the lowest mean cross-validation error. Variables remain in the model within the elastic net algorithm if the prediction error averaged over the tenfold cross-validation samples is reduced. In contrast to ordinary least squares regression or least absolute shrinkage and selection operator regularization, the elastic net algorithm performs well in highly correlated variables. It includes all variables with similar regression coefficients or excludes all variables from the best model. Regression coefficients of the model with 95% confidence intervals (CIs) were reported. Elastic net regularization was performed using the package *glmnet* version 4.1-3 in R version 3.6.2.

A principal component analysis with varimax rotation was performed to reduce the variation among healthcare providers among different factors.

All statistical tests were applied two-sided at a significance level of 0.05.

## Results

### Description of the Two Age Groups

Sociodemographic parameters and vertigo characteristics of the entire cohort and the two age groups are given in Table 1. Symptoms lasted longer in older adults than in younger persons Table 1). Compared with the younger group, older adults had a somatic reason for dizziness/vertigo more frequently. Older persons reported more falls related to dizziness/vertigo, however the effect size was small for this difference (Table 1). The amount of anxiety about bodily sensations (BSQ1) was higher in younger than in older adults (Table 2). However, both age groups did not differ in the BSQ2, which measures how often the sensations are experienced when the patient feels anxious or tense. Regarding emotional symptoms, older persons had fewer depressive symptoms and less anxiety according to the HADS and less vertigo-related maladaptive thoughts according to the adapted ACQ than younger individuals (Table 2). The two subscales computed from the adapted ACQ (physical crisis and loss of control) did not differ between both age groups (Table 2).

**Table 1:** Baseline characteristics of patients.

		**<60 years (*n* = 414)**	**≥60 years (*n* = 351)**	**All**	**Significance**
		**Mean ±SD**	**Mean ±SD**	**Mean ±SD**	
Age	Years	46.3 ± 9.6	71.2 ± 6.9	57.7 ± 15.0	*P* < 0.001, r = −1.0
Intensity of dizziness/vertigo	VAS 0–10	6.03 ±1.8	5.93 ± 1.8	5.1 ± 1.8	*P* > 0.05
Distress due to dizziness/vertigo	VAS 0–10	6.7 ± 2.0	6.3 ± 1.9	6.5 ± 2.0	*P* = 0.005, r = 0.11
		***N*** **(%)**	***N*** **(%)**	***N*** **(%)**	
Gender	Female	244 (58.9)	225 (64.1)	469 (61.3)	*P* > 0.05
	Male	170 (41.1)	126 (35.9)	296 (38.7)	
Medical diagnosis	BPPV	6 (1.5)a	17 (4.8)b	23 (3.0)	*P* < 0.001, r = 0.31
	BV	14 (3.4)a	21 (6.0)a	35 (4.6)	
	CV	12 (2.9)a	32 (9.1)b	44 (5.8)	
	MD	30 (7.3)a	25 (7.1)a	55 (7.2)	
	MultD	10 (2.4)a	110 (31.3)b	120 (46.8)	
	PPPD	269 (65.0)a	89 (25.4)b	358 (46.8)	
	VM	21 (5.1)a	9 (2.6)a	30 (3.9)	
	VN	43 (10.4)a	31 (8.8)a	74 (9.7)	
	VP	5 (1.2)a	5 (1.4)a	10 (1.3)	
	VS	4 (1.0)a	12 (3.4)b	16 (2.1)	
Diagnosis category	Somatic	145 (35.0)a	262 (74.6)b	407 (53.2)	*P* < 0.001, r = 0.38
	Non-somatic, psychogenic	233 (56.3)a	68 (19.4)b	301 (39.4)	
	Non-somatic, unspecific	36 (8.7)a	21 (19.4)a	57 (7.5)	
Permanent dizziness/vertigo	Yes	219 (56.6)	188 (56.8)	407 (56.7)	*P* > 0.05
	No	168 (43.4)	143 (43.2)	311 (43.3)	
Attacs	Yes	249 (67.5)	159 (52.3)	408 (60.6)	*P* < 0.001, r = −0.15
	No	120 (32.5)	145 (47.7)	265 (39.4)	
Falls	Yes	108 (28.5)	119 (37.2)	227 (32.5)	*P* = 0.017, r = 0.085
	No	271 (71.5)	201 (62.8)	472 (67.5)	
Disease duration	≤ 2 months	16 (4.0)a	5 (1.5)b	21 (2.9)	*P* < 0.001, r = −0.18
	2–6 months	53 (13.4)a	23 (7.0)b	76 (10.5)	
	0.5–1 years	93 (23.4)a	58 (17.6)a	151 (20.8)	
	1–2 years	95 (23.9)a	83 (25.2)a	178 (24.5)	
	3–5 years	66 (16.6)a	80 (24.2)b	146 (20.1)	
	>5 years	74 (18.6)a	81 (24.6)a	155 (21.3)	

*Significance refers to comparison between younger and older patients. Values in the same row and sub-table where the subscript is not identical differ strongly at p < 0.05 in the two-tailed test for equality for column proportions. The r indicates effect size by the Rank-Biserial correlation. SD, standard deviation; VAS, visual analog scale; BPPV, benign paroxysmal positional vertigo; BV, bilateral vestibulopathy; CV, central vertigo; MD, Meniere's disease; MultD, multisensory deficit; VM, vestibular migraine; VN, vestibular neuritis; VP, vestibular paroxysmia; VS, vestibular schwannoma*.

**Table 2:** Body Sensations Questionnaire (BSQ), Agoraphobic Cognitions Questionnaire (ACQ), and Hospital Anxiety and Depression Scale (HADS) in both age groups.

	**<60 years (*****n*** **= 414)**				**≥60 years (*****n*** **= 351)**			
	**Mean**	**SD**	**95% CI lower**	**95% CI upper**	**Mean**	**SD**	**95% CI lower**	**95% CI upper**
BSQ1	9.33_a_	5.60	8.77	9.89	6.72_b_	5.36	6.13	7.31
BSQ2	34.63_a_	74.38	27.12	42.14	33.20_a_	103.46	20.82	45.57
HADS anxiety	7.78_a_	4.09	7.38	8.18	5.71_b_	3.68	5.31	6.10
HADS depression	6.54_a_	4.05	6.14	6.94	5.75_b_	3.64	5.35	6.14
ACQ	20.61_a_	6.30	19.92	21.29	17.92_b_	4.44	17.28	18.57
ACQ physical crisis	1.60_a_	0.78	1.52	1.68	1.60_a_	0.81	1.50	1.70
ACQ loss of control	1.57_a_	0.82	1.48	1.65	1.56_a_	0.87	1.46	1.67

*Values in the same row and subtable where the subscript is not identical differ strongly at p < 0.05 in the two-tailed test for equality for column means. SD, standard deviation; CI, confidence interval; BSQ, Body Sensations Questionnaire; ACQ, Agoraphobic Cognitions Questionnaire; HADS, Hospital Anxiety and Depression Scale*.

### Healthcare Providers

**Figure 1:**
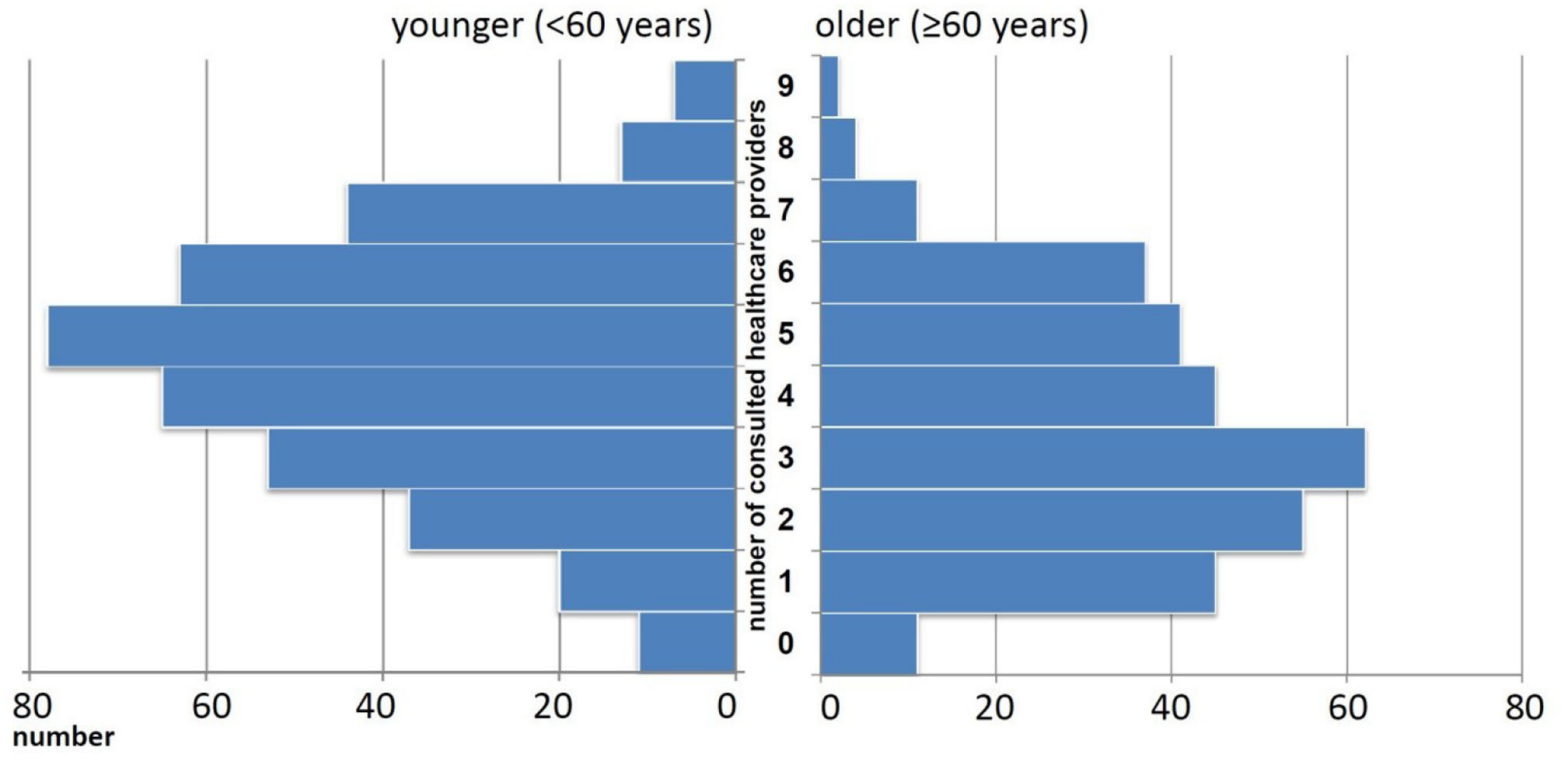
Number of different healthcare providers consulted for dizziness/vertigo.

**Figure 2:**
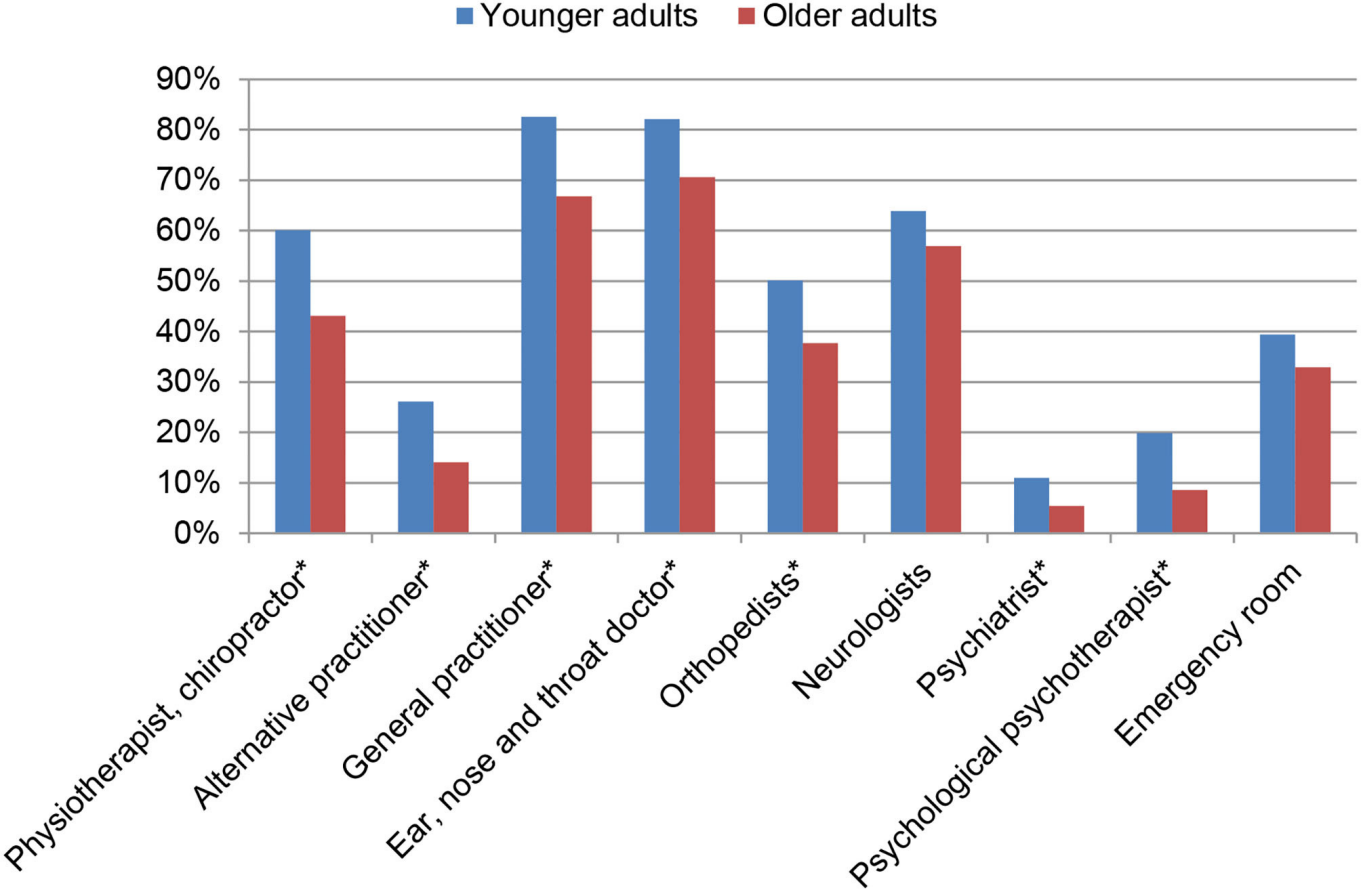
Utilization of medical assistance for dizziness in younger and older adults. * indicates significant group difference at *P* < 0.001.

In the previous year, most people saw more than one healthcare provider for dizziness/vertigo. On average, they consulted four ± two healthcare providers. The numbers of different healthcare providers consulted for dizziness/vertigo are displayed as histogram in Figure 1. In general, older adults indicated that they sought fewer healthcare providers for dizziness/vertigo (mean 3.4 ± 1.9, 95% CI 3.2, 3.6) than younger people (mean 4.5 ± 2.0, 95% CI 4.3, 4.7). This finding concerned both somatic (e.g., ENT specialist, orthopedist) and psychologically oriented healthcare providers (Figure 2). The different healthcare providers could be reduced to two factors: psychological and psychiatric loaded on one factor, and somatic-oriented healthcare providers loaded on a second factor (Table 3). Of the 681 people who consulted at least one healthcare provider, 396 (58.1%) consulted somatic-oriented and 285 (41.9%) psychologically oriented healthcare providers.

**Table 3:** Principal component analysis (PCA) of healthcare providers.

**Rotated component matrix**	**Factor 1**	**Factor 2**
Ear, nose and throat specialist	0.679	
General practitioner	0.640	
Orthopedist	0.619	
Physiotherapist/chiropractor	0.561	
Emergency unit		
Alternative practitioner		
Neurologist		
Psychiatrist		0.789
Psychologist		0.738
Eigenwert	2.391	1.147
% of variance	26.567	12.745
Cronbachs alpha	0.60	0.44

*Extraction method: Principal component analysis. Rotation method: Varimax with Kaiser normalization. Kaiser-Meyer-Olkin (KMO) = 0.76. Bartlett significance <0.001*.

People who consulted more healthcare providers were characterized by younger age, the presence of psychiatric reasons for dizziness/vertigo, a higher HADS-A, and a higher BSQ1 (Table 4). In the logistic regression, the likelihood of consulting somatic-oriented healthcare providers was higher for a lower ACQ and shorter disease duration of vertigo. However, the explained variance in this model was low (Table 5).

**Table 4:** Linear regression: predictors of consulted healthcare providers.

**Variable**	**Estimate**	**2.5% CI**	**97.5% CI**	***P***
(Intercept)	4.23	3.17	5.30	<0.001
Age	−0.02	−0.03	−0.00	0.01
Diagnosis category	0.34	−0.09	0.78	0.12
HADS anxiety	0.09	0.04	0.15	<0.001
BSQ1	0.02	−0.01	0.06	0.18

*Dependent variable: number of consulted healthcare providers. Independent variables: age, sex, diagnosis category [somatic vs. non-somatic (psychogenic and unspecific)], Hospital Anxiety and Depression Scale -Anxiety, Hospital Anxiety and Depression Scale -Depression, Agoraphobic Cognitions Questionnaire, Body Sensations Questionnaire-1, duration of vertigo (< or > 6 months). χ^2^(4) = 162.88, p = 0.00, Pseudo-R^2^ (Cragg-Uhler) = 0.12, AIC = 1444.10, BIC = 1467.28*.

**Table 5:** Binominal logistic regression: dependent variable consultation of somatic- (= 1) or psychologically (= 0) oriented healthcare provider (backward selection).

		**Coefficient**	**Standard error**	***P***	**Exp(B)**	**Nagelkerkes R^**2**^**	**Hosmer-Leme show test**
Step 1	Age	−0.011	0.007	0.119	0.989	0.028	0.109
	Sex	−0.118	0.203	0.560	0.888		
	Disease duration	−0.598	0.309	0.053	0.550		
	BSQ1	−0.010	0.019	0.608	0.990		
	HADS Anxiety	0.008	0.036	0.821	1.008		
	HADS Depression	−0.030	0.032	0.354	0.970		
	ACQ	−0.027	0.021	0.187	0.973		
	constant	3.041	0.852	0.000	20.927		
Step 2	Age	−0.012	0.007	0.103	0.989	0.027	0.186
	Sex	−0.123	0.202	0.544	0.885		
	Disease duration	−0.596	0.309	0.054	0.551		
	BSQ1	−0.009	0.019	0.634	0.991		
	HADS Depression	−0.026	0.028	0.348	0.974		
	ACQ	−0.026	0.020	0.189	0.975		
	Constant	3.059	0.849	0.000	21.299		
Step 3	Age	−0.011	0.007	0.107	0.989	0.027	0.208
	Sex	−0.116	0.202	0.566	0.891		
	Disease duration	−0.597	0.309	0.053	0.551		
	HADS Depression	−0.028	0.028	0.309	0.972		
	ACQ	−0.029	0.019	0.121	0.972		
	Constant	3.033	0.846	0.000	20.766		
Step 4	Age	−0.011	0.007	0.106	0.989	0.026	0.596
	Disease duration	−0.596	0.309	0.054	0.551		
	HADS Depression	−0.028	0.028	0.314	0.972		
	ACQ	−0.028	0.018	0.130	0.972		
	Constant	2.852	0.783	0.000	17.321		
Step 5	Age	−0.011	0.007	0.117	0.989	0.024	0.891
	Disease duration	−0.579	0.308	0.060	0.560		
	ACQ	−0.036	0.017	0.037	0.965		
	Constant	2.773	0.780	0.000	16.010		
Step 6	Disease duration	−0.639	0.305	0.036	0.528	0.018	0.544
	ACQ	−0.029	0.016	0.077	0.972		
	Constant	2.159	0.666	0.001	8.662		

*BSQ, Body Sensations Questionnaire; ACQ, Agoraphobic Cognitions Questionnaire; HADS, Hospital Anxiety and Depression Scale*.

### Hospitalizations

Overall, 285 (37.8%) individuals were hospitalized due to dizziness/vertigo in the year before their appointment at our Center for Vertigo and Dizziness. Younger people were more frequently hospitalized (*n* = 171, 24.4%) than older adults (*n* = 114, 16.3%) (*P* = 0.024). In the logistic regression, hospitalizations due to dizziness/vertigo were associated with younger age, a higher BSQ1, a higher HADS-D, and a lower HADS-A (Table 6). Results in the logistic regression did not change when additionally diagnosis category (somatic vs. non-somatic) was entered as independent variable.

**Table 6:** Logistic regression: predictors of hospitalization (dependent variable, backward selection).

		**Coefficent**	**Standard error**	***P***	**Exp(B)**	**Nagelkerkes R^**2**^**	**Hosmer-Lemeshow test**
Step 1	Age	−0.014	0.007	0.051	0.986	0.073	0.708
	Sex	−0.283	0.204	0.165	0.753		
	Disease duration	−0.437	0.275	0.112	0.646		
	BSQ1	0.061	0.020	0.003	1.063		
	HADS Anxiety	−0.093	0.036	0.010	0.911		
	HADS Depression	0.070	0.033	0.033	1.072		
	ACQ	−0.002	0.021	0.921	0.998		
	Constant	1.332	0.786	0.090	3.788		
Step 2	Age	−0.014	0.007	0.051	0.986	0.073	0.616
	Sex	−0.283	0.204	0.165	0.754		
	Disease duration	−0.439	0.274	0.110	0.645		
	BSQ1	0.060	0.019	0.002	1.062		
	HADS Anxiety	−0.094	0.034	0.006	0.910		
	HADS Depression	0.069	0.032	0.033	1.072		
	Constant	1.304	0.735	0.076	3.685		
Step 3	Age	−0.014	0.007	0.054	0.986	0.067	0.977
	Disease duration	−0.444	0.274	0.105	0.642		
	BSQ1	0.061	0.019	0.002	1.063		
	HADS Anxiety	−0.089	0.034	0.009	0.915		
	HADS Depression	0.067	0.032	0.038	1.069		
	Constant	0.871	0.662	0.188	2.388		
Step 4	Age	−0.015	0.007	0.030	0.985	0.060	0.732
	BSQ1	0.061	0.019	0.002	1.062		
	HADS Anxiety	−0.092	0.034	0.007	0.912		
	HADS Depression	0.070	0.032	0.030	1.073		
	Constant	0.139	0.482	0.773	1.149		

*BSQ, Body Sensations Questionnaire; ACQ, Agoraphobic Cognitions Questionnaire; HADS, Hospital Anxiety and Depression Scale. Disease duration (1 = <6 months, 2 = >6 months)*.

### Therapy

Older adults received less medication, less physiotherapy, and less psychological therapy for vertigo than younger people (Table 7).

**Table 7:** Therapy for vertigo/dizziness in both age groups.

		**<60 years**		**≥60 years**		***P***
**Treatment**		***N***	**%**	***N***	**%**	
Medication	No	162	40.3	171	49.7	0.010
	Yes	240	59.7	173	50.3	
Physiotherapy	No	192	47.8	203	59.0	0.002
	Yes	210	52.2	141	41.0	
Psychological therapy	No	320	79.6	304	88.4	
	Yes	82	20.4	40	11.6	0.001

## Discussion

Dizziness and vertigo are common complaints associated with a substantial healthcare burden (16, 17, 31, 32). Here, we analyzed three domains of healthcare utilization of patients attending a specialized vertigo center. For all studied healthcare utilization domains, we found age-dependent effects.

Approximately every second patient attending our vertigo center was at least 60 years old. In line with earlier studies, older patients had vertigo diagnoses with mainly degenerative or multifactorial etiologies, i.e., multisensory deficits or central vertigo (7, 23, 33). Taking the effect sizes of group comparison into account, one can summarize that the main difference between older and younger patients was found for the diagnosis category [somatic vs. non-somatic (psychogenic and unspecific)]. Somatic and multifactorial etiologies of dizziness were more common in older patients than younger patients who more frequently had non-somatic etiologies. This agrees with the observation that chronic conditions are often associated with balance dysfunction and dizziness in older patients (6). Older patients perceived slightly less distress due to dizziness than younger patients; however, the effect size was low to moderate. Older patients had less frequently attack-like dizziness. Anxiety about bodily sensations (BSQ1) was higher in younger patients than in older patients. Compared with younger patients, older patients reported fewer depressive and anxiety symptoms according to the HADS and less vertigo-related maladaptive thoughts according to the ACQ. Other studies showed an age-dependent decrease of the HADS in adults with vestibular neuritis (34). In a study of outpatients with otolaryngologic complaints, the age group between 30 and 50 years old had the highest scores for depression and was followed by anxiety in patients younger than 30 years old (35).

Vertigo and dizziness substantially contribute to population-attributable disability, especially during advanced age (36). In addition, major risk factors for falls can lead to fatal and nonfatal injuries, disability, and death (37, 38), escalating healthcare costs (39). Recurrent falls are often indicator events for admitting previously independent older people to long-term care institutions (14). Of note, 37% of our patients at least 60 years old reported falls due to dizziness and/or vertigo. This rate was higher than in the younger age group. Although, the effect size for different fall rate was low, this is aligned with existing data in community-dwelling individuals (40).

Although, when compared with younger individuals, older patients have somatic reasons for dizziness more frequently, experience more falls, and have similar distress due to dizziness, we observed remarkable differences in healthcare utilization between younger and older individuals.

First, the number of consultations with healthcare providers was considerably higher in younger patients than in older patients. Also, in the regression model, consultations with healthcare providers were associated with younger age patients after correcting for cofactors. General practitioners referred 48% of their patients with dizziness to at least one specialist. In 18% of cases, the specialist's diagnosis differed from the general practitioner's (41). In agreement with a large cohort of US-American patients who were ≥65 years old with balance disorders, most patients consulted multiple providers (42).

Second, hospitalizations due to dizziness and vertigo were associated with younger age after correcting for cofactors. We did not assess the reason for hospitalization (planned diagnostic stay vs. emergency). Therefore, one cannot conclude from a higher hospitalization rate on more serious pathology in younger adults. In our cohort, the hospitalization rate in the year before consultation at our outpatient clinic was relatively high. This is remarkable because our hospitalization rate is based on self-reports that are prone to recall bias and not on ICD 10-diagnosis based information. A study in Germany of 21 primary care practices demonstrated that 2% of patients with dizziness initially seen by a general practitioner were referred to a hospital because of severe symptoms or psychosocial reasons (41). The rate of inpatient treatment in ENT departments for acute dizziness in Thuringia (one of the 16 German federal states) was 54.71 per 100,000. Also, the incidence increased with age, with the highest incidence for patients 71–80 years old (43). In contrast, specialized centers for vertigo and dizziness generally treat dominantly chronic or refractory cases. They may, therefore, treat a higher number of patients with non-somatic syndromes, longer disease duration, and more frequent previous hospitalizations (23, 44).

Third, older patients in our study received less medication for dizziness/vertigo, less physiotherapy, and less psychological therapy for vertigo than younger patients. This agrees with a retrospective cohort study in patients referred to a tertiary care balance clinic in Germany, where older patients received fewer diagnostic and therapeutic measures and less medication than younger patients (45). Receiving less medication may not be a drawback *per-se*, as it was shown that, in many cases, potentially inappropriate drugs were prescribed in patients aged 70 and older (45). In addition, polypharmacy may be harmful in older patients with a detrimental effect on balance function (46). Many agents might contribute to the causes of dizziness than cure it in older adults (45, 47). In addition, anxiety and depression may play a minor role in older patients with dizziness/vertigo compared with younger patients (22). Therefore, healthcare providers may assume that psychological therapy may have less beneficial effects in older than in younger patients. However, with this assumption in mind, physiotherapy and occupational therapy should be primary therapeutic measures for dizziness/vertigo in the older population, which is not the case.

Due to the design of the study, we cannot explore the reasons for these differences in healthcare utilization in our study. We propose several suggestions. Diagnostic subgroups differ significantly regarding several diagnostic measures, therapies, and medications (45). One reason for these differences in healthcare utilization may be based on a different distribution of diagnoses, causing dizziness and vertigo between the age groups (7). Dizziness combined with anxiety is associated with increased subjective impairment and healthcare utilization (45). Anxiety, in contrast, was more pronounced in the younger age group. In addition, younger patients may consult healthcare services earlier, e.g., their capability to work.

Moreover, older patients may tolerate symptoms of dizziness and vertigo longer because patients, and possibly some medical doctors, may also believe that dizziness and balance disturbances may be age-associated phenomena (i.e., ageism). However, this is not true. Vertigo/dizziness is mainly not a consequence of normal aging of the vestibular and sensory systems (45). It is not adequate regarding the increasing burden of chronic and degenerative diseases and the increase in multifactorial origins of dizziness/vertigo in the older population. Especially regarding the increased risk of falls, and thereby, the increased danger of immobility and disability, the challenging target is a careful and comprehensive work-up of dizziness and vertigo in the older population.

As dizziness is often a heterogeneous disorder requiring interdisciplinary care, diagnostic accuracy must be improved with appropriate diagnostic testing to facilitate effective care plans for older patients with dizziness (45).

Our study has several limitations. Our sample of 765 patients with chronic dizziness/vertigo was physically and mentally independent. They consulted our specialized and multidisciplinary tertiary care outpatient clinic for dizziness and vertigo. Therefore, the results cannot be generalized to people with severe mobility impairments or cognitive deficits. Moreover, there is a selection bias regarding their underlying diagnoses. For example, only a few people had BPPV in our cohort, a common cause of dizziness among older adults (8). This is because today, general practitioners know how to diagnose, treat, and manage BPPV themselves. Patients are referred to our tertiary care center only if symptoms become chronic. As mentioned previously, due to the cross-sectional study design, we cannot make causal statements about the reasons for healthcare utilization differences. This must be addressed by qualitative and longitudinal studies.

## Data Availability Statement

The raw data supporting the conclusions of this article will be made available by the corresponding authors upon reasonable request for scientific purpose only.

## Ethics Statement

The studies involving human participants were reviewed and approved by Ethics Committee of the Friedrich-Schiller-University Jena, Jena, Germany. The patients/participants provided their written informed consent to participate in this study.

## Author Contributions

TP: design of the study and writing of the paper. HA and SF: collection of data. TP and HA: analysis. HA: revision of the paper. All authors contributed to the article and approved the submitted version.

## Conflict of Interest

The authors declare that the research was conducted in the absence of any commercial or financial relationships that could be construed as a potential conflict of interest.

## Publisher's Note

All claims expressed in this article are solely those of the authors and do not necessarily represent those of their affiliated organizations, or those of the publisher, the editors and the reviewers. Any product that may be evaluated in this article, or claim that may be made by its manufacturer, is not guaranteed or endorsed by the publisher.
